# Ire1-Mediated Unfolded Protein Response and Other Biological Phenomena in *Ascomycota* Yeasts and Filamentous Fungi

**DOI:** 10.3390/cells15201883

**Published:** 2026-10-15

**Authors:** Mohamad Aji Ikhrami, Yukio Kimata

**Affiliations:** 1 Division of Biological Science, Graduate School of Science and Technology, Nara Institute of Science and Technology, Nara 630-0192, Japan; mohamad.aji.ikhrami@mail.ugm.ac.id; 2 Department of Fisheries, Faculty of Agriculture, Universitas Gadjah Mada, Yogyakarta 55281, Indonesia

**Keywords:** endoplasmic reticulum, stress response, yeast, fungi, RNA

## Abstract

Upon dysfunction of the endoplasmic reticulum (ER), namely ER stress, eukaryotic cells commonly change their gene expression profile, resulting in enhanced ER functions. This cytoprotective response is called the unfolded protein response (UPR), whose molecular mechanism was first elucidated in pioneering studies using *Saccharomyces cerevisiae*. Ire1 is a transmembrane endoribonuclease that acts as an ER stress sensor. While Ire1-dependent unconventional splicing of mRNA encoding Hac1, the transcription factor responsible for UPR, was discovered in the 1990s, the precise mechanisms regulating their production and activation remain under investigation. Studies on Ire1 and Hac1 in other ascomycetes have also advanced, partly from the perspectives of clinical science and industrial applications, thereby uncovering some significant functional differences from those in *S. cerevisiae*. For instance, many ascomycete species other than *S. cerevisiae* likely perform regulated Ire1-dependent decay (RIDD), in which mRNAs encoding ER-client proteins are digested to mitigate ER stress. In certain species, Ire1 may preferentially shift its output toward *HAC1* mRNA splicing or RIDD, depending on the type of stimulus. This review article provides an overview of classic and recent findings on the regulation and function of Ire1 and its target mRNAs in various ascomycete species.

## 1. Introduction

The endoplasmic reticulum (ER) is a membrane-bound organelle commonly found in eukaryotic cells. It acts as the primary gateway for protein secretion and as a critical site for lipid biosynthesis [1,2]. Approximately one-third of all cellular proteins are folded and post-translationally modified in the ER before being transported to other organelles or the cell surface via vesicular transport. The ER thus provides a sophisticated environment to maintain the integrity of client proteins [2]. Cysteine disulfide formation and N-linked glycosylation are prominent protein modifications carried out in the ER.

To cope with the dysfunction or functional insufficiency of the ER, which frequently accompanies accumulation of unfolded proteins in the ER, eukaryotic cells commonly upregulate various genes, including those encoding ER-resident molecular chaperones and protein-folding enzymes. This phenomenon is known as the unfolded protein response (UPR), and conditions that trigger UPR are called ER stress. In addition to external stimuli or genetic mutations that disturb ER functions, high expression of ER-client secretory proteins can cause ER stress. While UPR was initially discovered in animal cells [3], its detailed mechanism has been elucidated through pioneering studies using the budding yeast, *Saccharomyces cerevisiae*, as a model organism.

**Figure 1:**
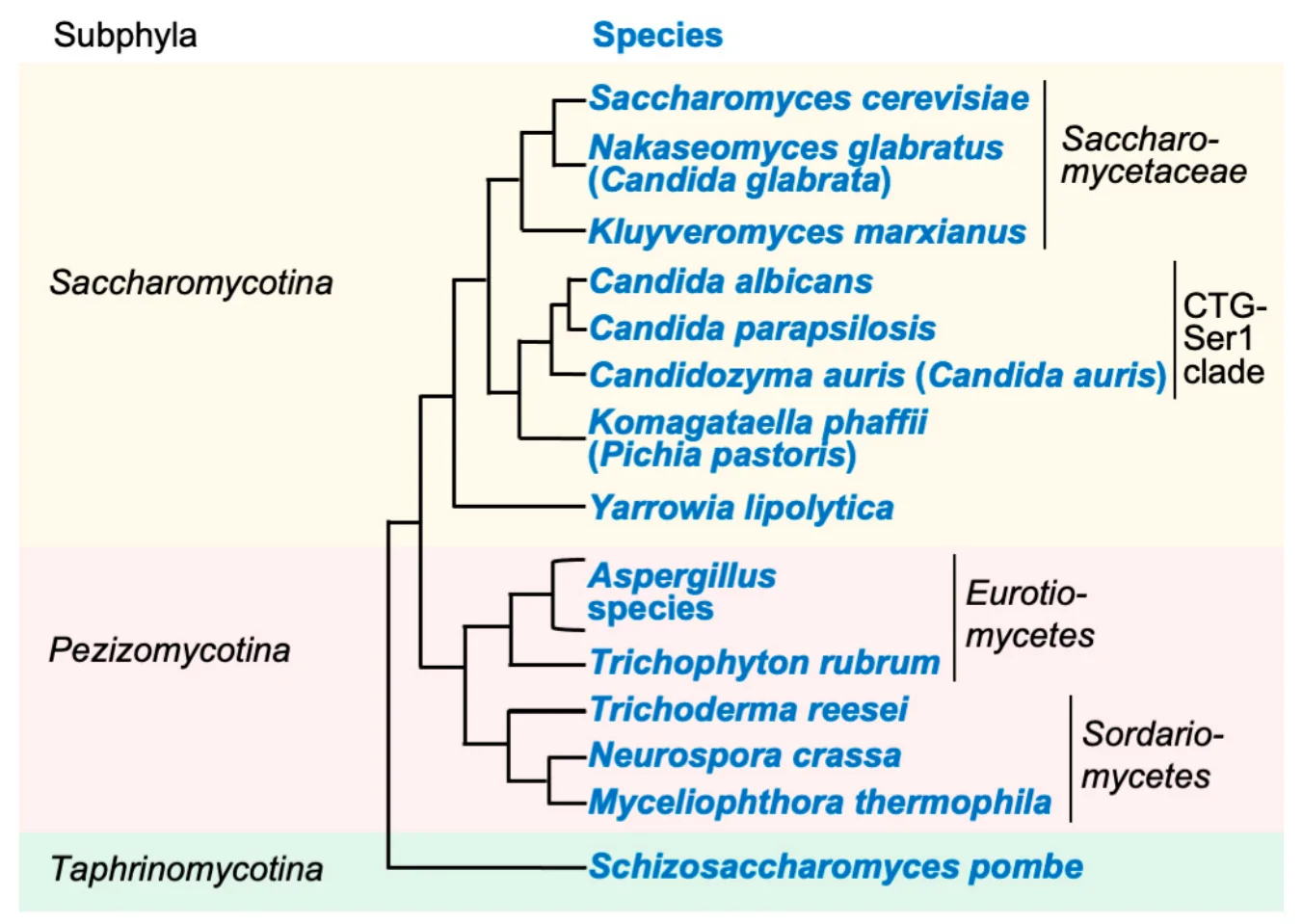
*Ascomycota* yeast and filamentous fungal species covered in this article. The phylogenetic relationship of the *Saccharomycotina* species is based on Ref. [4].

Ire1 is an ER-located transmembrane endoribonuclease that is conserved across eukaryotic organisms and acts as an ER stress sensor that triggers UPR. While Ire1 and its target, *HAC1* mRNA, were discovered in *S. cerevisiae* in the 1990s, new insights into their unique and intriguing properties are still emerging even today. Moreover, the functions and target molecules of Ire1 in other yeast and fungal species are not always identical to those in *S. cerevisiae*. In this review, we describe the fundamental and current insights into Ire1 and its target mRNAs in various ascomycete yeasts and filamentous fungi, whose phylogenetic relationship is shown in Figure 1.

## 2. Regulation and Function of Ire1 and Hac1 in *S. cerevisiae*

Through screening of UPR-deficient *S. cerevisiae* mutants, the Type-I transmembrane protein Ire1 was identified as a key UPR executor [5,6]. As described later, Ire1 triggers the UPR by splicing *HAC1* mRNA. To date, *HAC1* mRNA is regarded as the sole direct target of Ire1, at least in *S. cerevisiae* (Table 1). Consistent with this idea, Ref. [7] demonstrated that the *IRE1* and *HAC1* genes exhibit almost identical epistatic profiles.

Ire1 possesses Ser/Thr protein kinase and endoribonuclease motifs within its cytosolic domain. The luminal domain of Ire1 is responsible for its homo-association and higher-order oligomerization. As reviewed in Ref. [15], how the oligomeric status and activity of Ire1 are regulated in *S. cerevisiae* cells has been well explored. The prominent ER-resident molecular chaperone BiP associates with Ire1 to suppress its homo-association. Upon ER stress, BiP dissociates from Ire1, likely because unfolded proteins outcompete Ire1 for binding to BiP. Moreover, unfolded proteins accumulated in the ER lumen are directly captured by the luminal domain of Ire1 as activation ligands, leading to higher-order oligomerization of Ire1. The homo-association of Ire1 promotes its autophosphorylation, which is required for its activation as an endoribonuclease. In addition, ascomycete Ire1, unlike animal or plant orthologs, bears an intrinsically disordered segment at its N-terminus to suppress the activation of Ire1 under non-stress conditions. It should also be noted that ADP is accommodated in the catalytic center of the kinase subdomain of Ire1, significantly enhancing its endoribonuclease activity [16,17]. Severe ER stress may damage cells, leading to impaired ATP biosynthesis and elevated cytosolic ADP levels, which in turn results in strong UPR induction [18]. Disturbance of the membrane-lipid composition, namely lipid bilayer stress (LBS), represents another type of ER stress that activates Ire1 and induces the UPR independently of the accumulation of unfolded proteins in the ER lumen [19,20].

Elevated expression of Ire1 also upregulates the UPR in *S. cerevisiae*. Ref. [21] recently proposed that, under ER stress, the MAP kinase Slt2 phosphorylates and activates the transcription factor Rlm1, which then transcriptionally induces Ire1 to boost UPR.

**Figure 2:**
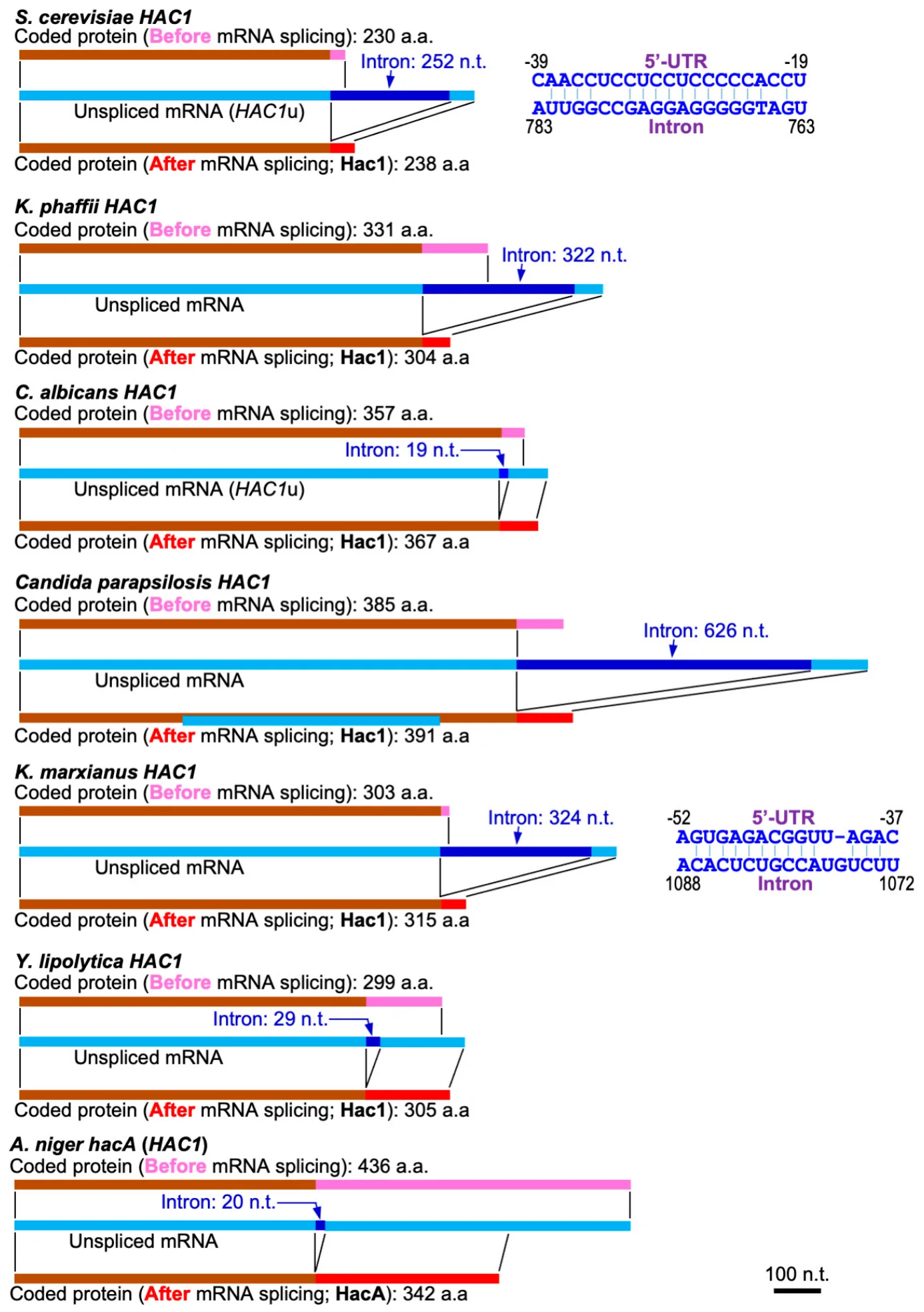
*HAC1* mRNA structures across various *Ascomycota* species. The middle line of each trio indicates *HAC1* mRNA not spliced by Ire1. The dark blue part represents the intron excised via unconventional splicing during ER stress. The light blue parts represent putative protein-coding regions of the first and second exons, while the 5′- and 3′-UTRs are omitted. The upper line indicates the predicted protein product encoded by the unspliced *HAC1* mRNA. After splicing, *HAC1* mRNA is predicted to be translated into the protein indicated by the bottom line, which is generally called Hac1. This unconventional splicing alters the C-terminal sequence of the encoded proteins, as indicated in pink and red. The base-pairing interaction between the 5′-UTR and intron of *S. cerevisiae* unspliced *HAC1* mRNA [25] is also shown in this figure. Unspliced *HAC1* mRNA of *K. marxianus* likely carries a similar structure.

Hac1, a bZIP-type transcription factor responsible for the induction of UPR target genes, was also initially identified in *S. cerevisiae* [22]. *S. cerevisiae* cells have two types of *HAC1* mRNA, namely *HAC1*u (“uninduced”) and *HAC1*i (“induced”). The *HAC1*u mRNA is the transcript of the *HAC1* gene and bears an intron sequence (Figure 2). Upon ER stress, Ire1 cleaves the two exon-intron junctions of the *HAC1*u mRNA, and the tRNA ligase Trl1 (alias Rlg1) fuses the two exon fragments in the cytoplasm to yield the intronless *HAC1*i mRNA [23]. The mechanism of this splicing reaction [24], which is called unconventional splicing, is entirely distinct from that of the canonical pre-mRNA splicing in the nucleus.

As shown in Figure 2, the translation product from *HAC1*i mRNA (Hac1) and that from *HAC1*u mRNA have different C-terminal sequences. Albeit weakly, the translation product from *HAC1*u mRNA is functional, as its overexpression partially rescued UPR-deficient phenotypes of *ire1Δ* cells in *S. cerevisaie* [22]. However, at least in *S. cerevisiae*, protein production from the *HAC1*u mRNA is severely repressed. Ref. [25] showed that intramolecular base-pairing between the 5′-untranslated region (UTR) and intron (Figure 2) inhibits the translation of *HAC1*u mRNA. According to Refs. [26,27], this base-pairing inhibits the translation initiation of the *HAC1*u mRNA. Furthermore, the leaky translation product of *HAC1*u mRNA is rapidly degraded by the ubiquitin-proteasome system, since it has a degron sequence at the C-terminus [28].

Refs. [29,30] demonstrated that, for effective splicing by Ire1, *HAC1* mRNA is recruited to Ire1 foci, which are formed by the high-order oligomerization of Ire1 upon ER stress. The 3′-UTR of *HAC1* mRNA forms a unique stem-loop structure containing a conserved bipartite element, which is required for its colocalization with Ire1 [29]. Pal1 is a poorly characterized protein known to localize to the endocytic sites [31]. According to Ref. [32], Pal1 and its paralog Pal2 bind to the 3′-UTR of *HAC1* mRNA, thereby facilitating its recruitment to Ire1 foci. Kin1 and its paralog Kin2 are Ser/Thr protein kinases thought to be involved in several biological processes in the secretory pathway [33]. They also promote the colocalization of Ire1 and *HAC1* mRNA via Pal2 phosphorylation [32,34].

According to Ref. [35], ER stress induces the monoubiquitination of the ribosomal protein eS7, thereby upregulating *HAC1* mRNA translation. Ref. [36] demonstrated that this phenomenon results from the degradation of the deubiquitinase by Grr1. TOR is a Ser/Thr protein kinase that acts as a master regulator of cell growth and metabolism in response to nutrient availability [37]. Gcn2 is a Ser/Thr protein kinase that attenuates global protein synthesis upon amino acid starvation [38]. Translation of *HAC1* mRNA is also promoted by TOR and Gcn2, as well as by the phosphatidylinositol-3-kinase Vps34 [39], suggesting coordination between UPR and other intracellular signaling pathways that respond to nutrient conditions.

Hac1 transcriptionally induces the *HAC1* gene, suggesting a positive feedback mechanism that amplifies and sustains UPR in *S. cerevisiae* cells [40]. In contrast, *HAC1* mRNA is likely to be negatively regulated to attenuate UPR following its peak induction. According to Ref. [41], nuclear protein Isw1 is induced by ER stress and binds to *HAC1* mRNA, which is then retained in the nucleus. Recently, it has been proposed that the downregulation of the nuclear RNA exosome suppresses *HAC1* mRNA splicing, possibly due to the accumulation of unprocessed RNA species [42]. However, the nuclear RNA exosome was previously reported to directly trim *HAC1* mRNA, which then lacks the 3′-UTR and is poorly spliced, under unstressed conditions [43,44].

Under harsh stress conditions that halt global protein synthesis, *HAC1* mRNA splicing does not lead to UPR gene induction because *HAC1*i mRNA is not translated into Hac1 [45]. This phenomenon is likely physiologically meaningful, as the ER protein folding capacity may not need to be enhanced in the absence of nascent protein influx.

The UPR was initially known to induce genes encoding BiP, BiP cofactors, and ER-resident protein folding enzymes, such as protein disulfide isomerase (PDI). However, transcriptome analyses of *S. cerevisiae* cells have revealed that a broader range of genes are upregulated depending on the *IRE1* and *HAC1* genes during ER stress [46,47]. UPR target genes induced by Hac1 include various genes involved in the protein secretory pathway, such as ER protein translocation, protein folding, disulfide-bond formation, glycosylation, and vesicle transport. It is noteworthy that Hac1 binds to two different promoter DNA motifs in distinct modes, thus explaining the broad range of its target genes [48].

ER-associated protein degradation (ERAD), a process by which unfolded proteins accumulated in the ER are retrotranslocated to the cytosol for proteasomal degradation [2,49], is upregulated by UPR through the transcriptional induction of ERAD factors [46]. Furthermore, by inducing lipid biosynthesis genes, UPR drives ER expansion, which also helps mitigate ER stress [50]. According to Ref. [51], the set of UPR-induced genes under LBS differs from that induced by ER proteotoxic stress. This insight suggests that the function of Hac1 is modulated according to the type of stress.

**Figure 3:**
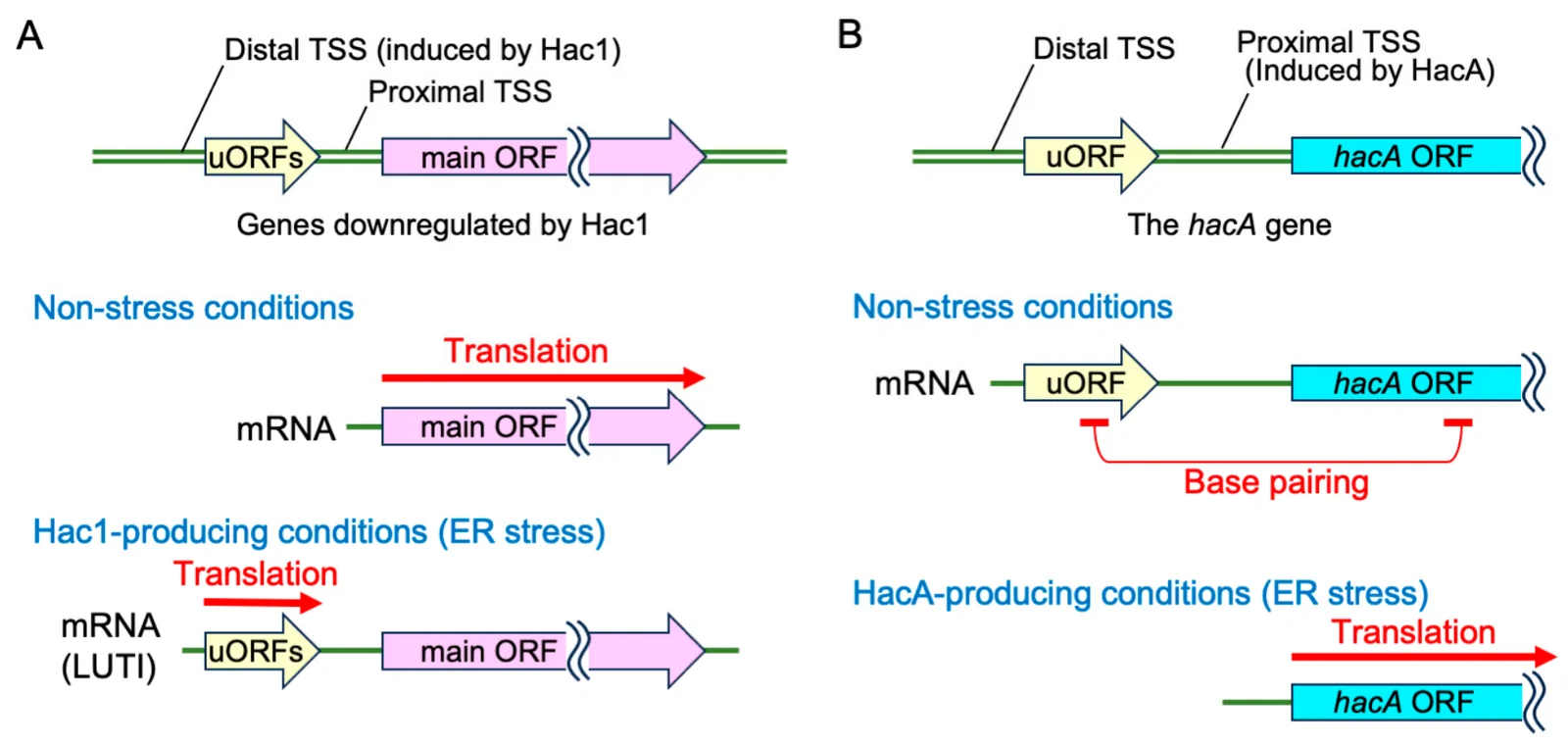
Shifting the TSSs by Hac1. (**A**) Hac1 produces LUTIs from several genes in *S. cerevisiae*. Under non-stress conditions, these genes are normally transcribed and translated. In contrast, Hac1 yields the LUTIs, from which uORFs are translated, resulting in the translational suppression of the main ORFs [52,53]. (**B**) Under non-stress conditions, *A. niger hacA* mRNA possesses a long 5′-UTR that contains uORFs and forms an intramolecular base-pairing, leading to its translational suppression. In contrast, HacA yields shorter 5′-UTR variants that are effectively translated.

Hac1 also suppresses the expression of certain genes in *S. cerevisiae* cells. It has been proposed that UPR downregulates genes encoding cell-surface transmembrane proteins, thereby decreasing the entry of nascent peptides into the ER and mitigating ER stress [47]. Two publications demonstrated the involvement of long undecoded transcript isoforms (LUTIs) in Hac1-dependent gene suppression [52,53]. As illustrated in Figure 3A, a gene containing a Hac1-driven distal transcription start site (TSS) and upstream open reading frames (uORFs) yields a LUTI, which is not translated into a protein. According to Ref. [52], this event contributes to the downregulation of the electron transport chain upon ER stress.

The Ire1 and *HAC1*-dependent UPR signaling pathway has been reported to be activated by various stress stimuli. The N-glycosylation inhibitor tunicamycin and the disulfide-reducing agent dithiothreitol (DTT) are widely used ER stressors that activate Ire1, presumably through the accumulation of aberrant proteins in the ER. In contrast, inositol depletion triggers the UPR by eliciting LBS [19]. Exposure to ethanol, cadmium, or acetic acid has also been shown to activate the UPR signaling pathway [54,55,56,57,58,59]. Deleting the *IRE1* or *HAC1* gene results in hypersensitivity to these stress stimuli. Ref. [60] reported that *HAC1* mRNA is more strongly spliced under ethanol stress in industrial ethanol-fermentation strains than in the laboratory strain.

Nevertheless, it is also likely that the UPR supports the growth and survival of *S. cerevisiae* cells even without external stress stimuli. Ref. [61] reported that UPR is transiently induced upon the diauxic shift, when cells robustly activate mitochondrial respiration after the consumption of fermentable sugars. The UPR signaling pathway has also been shown to extend the chronological lifespan [62]. According to Ref. [63], deletion of the *IRE1* or *HAC1* gene is likely to severely retard cell growth, depending on the strain background. Notably, this growth delay was rescued by aneuploidy of specific chromosomes, which presumably mitigated proteotoxic stress in the ER [63]. The baseline UPR under unstressed conditions thus maintains ER proteostasis integrity, which, according to Ref. [64], is important for cytokinesis. During cytokinesis, various proteins are transported to the bud neck via the secretory pathway, possibly causing overload of the ER, thereby inducing ER stress.

Taken together, in *S. cerevisiae* cells, various stress stimuli cluster and activate Ire1, directly inducing *HAC1* mRNA splicing, which results in the upregulation or downregulation of a number of genes. The production and activity of Ire1 and Hac1 are regulated in a complex manner, presumably to elicit UPR at the appropriate timing and magnitude.

When Hac1 is artificially and constitutively expressed in *S. cerevisiae* cells, many of the UPR-target genes are transcriptionally induced, resulting in the ER expansion [65,66]. Constitutive expression of Hac1 has been reported to increase the secretion yields of endogenous and heterologous recombinant proteins [66,67]. Ref. [68] demonstrated the positive effect of co-expression of Hac1 with BiP or PDI on heterologous ovalbumin secretion. Genetic manipulations that likely support Hac1 production, such as the overexpression of Kin1 or Pal2, have also been shown to increase heterologous protein secretion [69]. Another strategy for enhancing heterologous protein secretion involves modifying the Hac1-responsive promoter of a UPR target gene to boost its expression [70]. According to Ref. [71], constitutive expression of Hac1 improved the quality of recombinant insulin secreted from *S. cerevisiae* cells. In addition to the protein secretion, heterologous terpenoid and carotenoid production can be increased by the artificial expression of Hac1 [65,66,72,73].

These insights highlight the potential of UPR modulation for the high-yield production of commercially valuable biomolecules. However, a key limitation of this strategy is that constitutive and artificial UPR activation severely retards cell growth [65]. To avoid this problem, Hac1 was expressed under the control of the regulatable Tet-off promoter [73]. Furthermore, fast-growing and high-UPR mutants were isolated [66]. Ref. [66] reported that histone deacetylase A-deficient mutants grew well even during constitutive Hac1 expression.

## 3. Function of Ire1 in *Schizosaccharomyces pombe*

Like *S. cerevisiae*, *S. pombe* (fission yeast) is an *Ascomycota* yeast widely used as an established model organism in molecular and cellular biology. These two species are evolutionarily divergent, as *S. cerevisiae* and *S. pombe* belong to the subphyla *Saccharomycotina* and *Taphrinomycotina*, respectively (Figure 1). While Ire1 is evolutionally conserved, *S. pombe* lacks a *HAC1* ortholog [8,74]. *S. pombe* Ire1 is thus unlikely to be involved in mRNA splicing (Table 1). This is noteworthy because many other eukaryotes contain bZIP-family transcription factors, such as animal Xbp1 and plant bZIP60, whose mRNAs are subjected to Ire1-driven unconventional splicing [23,75].

Ire1 is thought to solely perform regulated IRE1-dependent decay (RIDD) in *S. pombe* [8] (Table 1). RIDD, which was initially found in Drosophila [76], has been reported to occur in a wide variety of eukaryotes, including plants and other animals [75,77]. In RIDD, Ire1 cleaves specific RNA molecules in response to ER stress, leaving them unligated. A prominent role of RIDD is the degradation of mRNAs encoding ER client proteins, thereby presumably mitigating ER stress by reducing nascent protein influx into the ER. However, Ire1 has also been reported to cleave microRNAs that translationally repress the expression of a pro-apoptotic caspase, leading to apoptosis under prolonged ER stress, in mammalian cells [78].

Transcriptome analysis has shown that many genes encoding ER client proteins are downregulated depending on Ire1 and ER stress in *S. pombe* [8]. Transcript mRNAs from these genes are likely to be cleaved by Ire1 and then digested by the Ski2-containing cytosolic exosome. Ref. [8] reported that cleavage fragments of a RIDD-substrate mRNA were detected in *ski2Δ* cells but not in wild-type, *ire1Δ*, or *ire1Δ*/*ski2Δ* cells. No-go decay (NGD) is an mRNA quality control system in which ribosome stalling triggers mRNA degradation [79]. According to Ref. [80], when Ire1 cleaves mRNA within its ORF, the fragments undergo NGD, thereby mitigating ER stress. Intriguingly, BiP mRNA is cleaved within its 3′-UTR by RIDD, resulting in its stabilization and the induction of BiP [8]. The RIDD substrate mRNAs likely contain a consensus motif recognized and cleaved by Ire1 [8,80].

ER-phagy, which contributes to the alleviation of ER proteotoxic stress, is a selective autophagic process that degrades portions of the ER in lysosomes or vacuoles [81]. *S. pombe* Epr1, a key factor in ER-phagy, is induced in response to ER stress in an Ire1-dependent manner [82]. However, unlike BiP mRNA, Epr1 mRNA does not appear to be a direct target of RIDD [82].

According to Ref. [83], substrate RNA specificity differs between *S. cerevisiae* and *S. pombe* Ire1, thereby explaining the functional divergence of these two Ire1 orthologs in *HAC1* mRNA splicing and RIDD. Biochemically, as an endoribonuclease, *S. pombe* Ire1 appears to be less stringent in substrate recognition than its *S. cerevisiae* ortholog. Ref. [84] proposed a structural biological basis for the difference in substrate specificity.

Taken together, in *S. pombe*, Ire1 does not splice mRNA encoding the bZIP-family transcription factor. Instead, many mRNAs encoding ER-client proteins are degraded by RIDD, likely mitigating the protein load on the ER. However, BiP is induced during ER stress because its mRNA is stabilized by RIDD.

## 4. Function of Ire1 and *HAC1* in *Komagataella phaffii*

*Pichia pastoris*, now reclassified as *Komagataella phaffii* or *Komagataella pastoris*, is a methylotrophic yeast species that belongs to the subphylum *Saccharomycotina*, as does *S. cerevisiae* (Figure 1). Several publications have shown that, in *K. phaffii*, Ire1 promotes splicing of *HAC1* mRNA, whose structure is presented in Figure 2 [85,86,87]. This leads to the transcriptional induction of various genes, including those involved in ER protein translocation, folding, and modification, as seen in *S. cerevisiae* [9,88]. It is also likely that expression of the *HAC1* gene is upregulated by Hac1 in a positive feedback loop, as well as in *S. cerevisiae* [9,87]. Unlike *S. cerevisiae*, *K. phaffii* exhibits efficient *HAC1* mRNA splicing even under non-stress conditions, presumably due to its high protein secretion capacity [85,87]. Moreover, *HAC1* mRNA splicing is further and considerably upregulated when cells are cultured at high temperatures in *K. phaffii*, whereas heat stress induces *HAC1* mRNA splicing only slightly in *S. cerevisiae* [9,89].

Another intriguing difference from *S. cerevisiae* is that in *K. phaffii*, the *ire1Δ*/*hac1Δ* double-deletion mutant grew more slowly than the *ire1Δ* or *hac1Δ* single mutants [9]. This observation indicates that, in *K. phaffii*, the *IRE1* and *HAC1* genes have mutually independent functions. It is thus possible that the unspliced form of *HAC1* mRNA, in addition to the spliced form, is translated to produce a functional protein. According to Ref. [90], the C-terminal degron sequence of the unspliced *HAC1* mRNA translation product is unlikely to be conserved in *K. phaffii*. Ref. [9] also demonstrated that deletion of the *IRE1* gene, but not of the *HAC1* gene, disturbed cytosolic protein integrity, suggesting an undiscovered role of Ire1 (Table 1).

*K. phaffii* is widely used as a platform for producing valuable recombinant proteins, including biopharmaceuticals, which are frequently secreted into the culture medium [91,92]. To improve the yield of secretory proteins, extensive efforts have focused on engineering the protein secretory pathway, which begins at the ER [91]. As reviewed in [93], many studies have shown that Hac1 overexpression enhances the secretory yield of recombinant proteins in *K. phaffii*. It has also been reported that overexpression of BiP and PDI—which are induced by the Ire1- and *HAC1*-dependent UPR in wild-type cells—increases the secretory protein yield [93]. Although these strategies are not always highly effective, they are promising and continue to be used in very recent publications [94,95,96]. According to Ref. [97], the secretion of recombinant antibody fragments was considerably elevated by co-overexpressing Hac1 and another stress-responsive transcription factor, Msn4.

The effect of Hac1 overexpression on increasing secretory yield is highly dependent on the nature of the target proteins [93]. Moreover, to achieve a high yield of secretory proteins, the timing and level of Hac1 expression are important factors. For instance, constitutive overexpression of Hac1 lowered the secretory yield of a test protein, which conversely, was improved when Hac1 was expressed under a regulated promoter [98]. Constitutive expression of Hac1 may exert adverse effects on *K. phaffii* cells. As exemplified in the previous paragraph, the co-expression of Hac1 with other proteins that also improve protein secretion is likely to be an effective and promising strategy [93]. As demonstrated in Ref. [99], it may also be possible to improve the protein secretory yield by the expression of heterologous transcription factors, such as human Xbp1, instead of native *K. phaffii* Hac1.

Taken together, as in *S. cerevisiae*, Ire1 promotes *HAC1* mRNA splicing to upregulate the protein-folding capacity of the ER in *K. phaffii*. Consequently, the secretory yield of heterologous recombinant proteins can be increased by overexpressing Hac1. However, unlike *S. cerevisiae*, *IRE1* and *HAC1* are also likely to play distinct roles in *K. phaffii*.

## 5. Function of Ire1 and *HAC1* in *Candida* and Related Species

*Candida* species, which also belong to the subphylum *Saccharomycotina* (Figure 1), are generally known as infectious yeasts. Several publications have described the roles of Ire1 and Hac1 in *Candida albicans*. It has been clearly demonstrated that, as in *S. cerevisiae*, Ire1 promotes *HAC1* mRNA splicing under ER stress conditions in *C. albicans*, contributing to ER stress tolerance [10,100,101] (Table 1).

Several factors have been reported to modulate the Ire1/*HAC1*-dependent UPR signaling pathway in *C. albicans*. According to Ref. [102], *IRE1* and *HAC1* gene expression is upregulated upon heat stress by the signaling mucin Msb2, presumably through the activation of the CEK mitogen-activated proteins kinase and/or the protein kinase C pathway. Ref. [103] demonstrated that deletion of the *UPC2* gene, which encodes a key transcription factor regulating ergosterol biosynthesis, attenuated *HAC1* mRNA splicing. In *C. albicans*, as in *S. cerevisiae,* the high-osmolarity glycerol (HOG) MAPK signaling pathway is activated upon ER stress [104,105]. The HOG signaling pathway attenuates *HAC1* mRNA splicing, likely by alleviating ER stress [104]. Ref. [104] also indicated that, under ER stress, Ire1 promotes the phosphorylation of Hog1, a marker of HOG pathway activation.

As a topic specific to *C. albicans*, the *IRE1* and *HAC1* genes support hyphal development and pathogenesis, consistent with their roles in maintaining the proper folding, modification, and transport of secretory and cell-surface proteins [10,100,101,106]. Ref. [107] has shown that the Ire1/*HAC1*-dependent UPR signaling pathway is involved in cellular tolerance to manganese toxicity. Because of its pathogenesis, the mode of action of antifungal drugs is important in *C. albicans* biology. Fluconazole, monoterpene carvacrol, and tetrahydrocarbazole CAR8 have been shown to trigger *HAC1* mRNA splicing, which likely contributes to cellular tolerance to these chemicals [103,108,109]. In other words, these chemicals somehow induce ER stress, which damages cells. Given the involvement of Ire1 and/or Hac1 in pathogenesis, their chemical inhibitors could also be used for antifungal therapies. Ref. [110] demonstrated that a prominent Ire1 inhibitor, 4μ8C, effectively suppresses *C. albicans* infectivity.

The intron sequence of *C. albicans HAC1* mRNA, which is removed by Ire1, is considerably shorter than that of the *S. cerevisiae* ortholog [74,100] (Figure 2). The ORF of *C. albicans HAC1* mRNA extends into the second exon even before splicing (Figure 2). However, similar to animal Xbp1 mRNA, Ire1-mediated splicing of *HAC1* mRNA causes a frameshift, resulting in different C-terminal sequences between the proteins encoded by its unspliced and spliced forms (Figure 2). The intron seems too short to suppress *HAC1* mRNA translation via its intramolecular hybridization to the 5′-UTR [74]. On the other hand, ER stress also results in the truncation of the 5′-UTR of *HAC1* mRNA, which may affect its secondary structure and translation efficiency [100].

It should also be noted that, unlike in *S. cerevisiae*, Ire1 in *C. albicans* has functions other than *HAC1* mRNA splicing (Table 1). Ref. [10] demonstrated that the *IRE1* gene, but not the *HAC1* gene, is involved in cellular growth under iron-restricted conditions. Moreover, *HAC1* knockout impaired hyphal development, but to a lesser extent than *IRE1* knockout [11]. According to Ref. [101], although *IRE1*-deficient cells were highly sensitive to drugs disrupting cell wall and membrane homeostasis, these chemicals failed to induce *HAC1* mRNA splicing in wild-type cells. Hyphal development is also unlikely to trigger *HAC1* mRNA splicing strongly [11]. Intriguingly, transcriptome data presented in Ref. [11] revealed that ER proteotoxic stress and hyphal development induce distinct sets of genes in Ire1-dependent manners. Ref. [10] have shown that the *IRE1* gene, but not the *HAC1* gene, contributes to the growth of *C. albicans* under high-temperature conditions. Taken together, these insights indicate that Ire1 differentially activates *HAC1*-dependent and -independent pathways in *C. albicans*, according to the nature of stress. Ref. [11] also suggested that Ire1 carries out RIDD in *C. albicans*.

As shown in Ref. [111], many other yeast species belonging to the CTG-Ser1 clade [112], as well as *C. albicans*, are predicted to possess *HAC1* mRNA with introns of diverse lengths that can be removed by Ire1-dependent unconventional splicing. Ref. [111] also presented experimental data indicating that the long intron of *Candida parapsilosis* is removed upon ER stress (Figure 2), presumably inducing various genes, including those involved in ER protein homeostasis. Ref. [113] demonstrated splicing of *HAC1* mRNA in *Candidozyma auris* (basionym *Candida auris*), which likely contributes to ER stress tolerance and virulence.

## 6. Function of Ire1 and *HAC1* in Other *Saccharomycotina* Yeasts

According to molecular phylogenetic analysis, *Candida glabrata* has been reclassified into another genus that is phylogenetically close to *Saccharomyces* [114] and renamed as *Nakaseomyces glabratus* (Figure 1). In common with other *Saccharomycotina* species, *N. glabratus* has the *IRE1* and *HAC1* genes. However, in *N. glabratus*, *HAC1* mRNA is not spliced even under ER stress conditions, and another signaling pathway governs transcriptional induction in UPR [12]. *N. glabratus* Ire1 is proposed to mitigate ER stress not through *HAC1* mRNA splicing, but rather through RIDD [12] (Table 1).

*Kluyveromyces marxianus*, a member of the family *Saccharomycetaceae* along with *S. cerevisiae* (Figure 1), is known as a thermotolerant yeast species [115]. Ref. [116] demonstrated that, in *K. marxianus*, conventional ER stress stimuli caused by tunicamycin or DTT trigger *HAC1* mRNA splicing (Figure 2), presumably promoted by Ire1 and leading to the transcriptional induction of genes involved in ER functions. As shown in Figure 2, the unspliced form of *K. marxianus HAC1* mRNA possibly forms an intramolecular base-pairing between the 5′-UTR and intron, which may contribute to its translation inhibition, similar to *S. cerevisiae*. Given the high thermotolerance of *K. marxianus*, it should be highly worthwhile to elucidate the roles of Ire1, Hac1, and UPR in its survival and growth at high temperatures.

Another *Saccharomycotina* yeast, *Yarrowia lipolytica*, has been well studied due to its oleaginous nature [117]. *HAC1* mRNA of *Y. lipolytica* has a short intron that is excised in response to ER stress, as well as *C. albicans* [118] (Figure 2). Considering the observations in Refs. [118,119], it is highly plausible that Ire1 conducts the splicing of *HAC1* mRNA to mitigate ER stress in *Y. lipolytica*, as observed in *S. cerevisiae*. Deletion of the gene encoding the BiP cofactor Sls1 has been shown to impair BiP association with Ire1 and trigger the UPR, suggesting a role for Sls1 in UPR regulation [119]. Ref. [120] demonstrated that *HAC1* overexpression enhances protein secretion in *Y. lipolytica*.

## 7. Function of Ire1/IreA and Hac1/HacA in Ascomycete Filamentous Fungi

A number of filamentous fungal species, including *Aspergillus*, belong to the subphylum *Pezizomycotina* (Figure 1). Based on standard nomenclature, the *IRE1* and *HAC1* gene orthologs in *Aspergillus* and related species are designated as *ireA* and *hacA*, respectively. The *Pezizomycotina* fungi likely possess a UPR machinery that is fundamentally similar to that of *Saccharomycotina* yeasts. Several publications have demonstrated that ER stress causes splicing of *hacA* (or *HAC1*) mRNA, which is then translated into HacA (or Hac1), likely leading to the transcriptional induction of genes involved in protein folding and modification in the ER [121,122,123,124,125,126,127,128,129]. While the *hacA* (or *HAC1*) transcript undergoes conventional splicing to become mRNA in *Pezizomycotina* fungi, we do not address this process here. Unlike that of *S. cerevisiae HAC1* mRNA, the *hacA* intron sequence excised via the unconventional splicing is only 20-nt long in various *Aspergillus* species and *Trichophyton rubrum* [121,122,123,124,125,126] (Figure 2). In the *Sordariomycetes*, the intron lengths are 20 nt in *Trichoderma reesei*, and 23 nt in *Neurospora crassa* and *Myceliophthora thermophila* [121,127,130]. The amino acid sequence encoded by the second exon of unspliced *hacA* mRNA (the pink segment in Figure 2) is remarkably long and highly conserved among *Aspergillus* species [122]. This implies that, in addition to HacA, the translation product of unspliced *hacA* mRNA may have a biological function, as experimentally supported in Ref. [131].

As depicted in Figure 3B, *hacA* mRNA has a long 5′-UTR, which causes its translational repression, in non-stressed *Aspergillus niger* cells [132]. On the other hand, the 5′-UTR of the *hacA* gene contains HacA-binding sites, and its TSS shifts downstream upon HacA production [122,129,132]. This results in the translational induction of the *hacA* gene during ER stress. The same is likely to be said for *Aspergillus nidulans* and *T. reesei* [129].

Many filamentous fungal species exhibit high levels of glycoside hydrolase and protease secretion, which often makes them highly advantageous for industrial applications. Overexpression of HacA has been shown to increase the secretory yield of heterologous proteins in *A. niger* [133]. The same is likely to be said for *T. reesei* [134]. When hydrolytic enzyme secretion is induced in *Aspergillus oryzae*, *hacA* mRNA undergoes splicing, which likely supports cellular growth [135]. According to Ref. [130], Hac1 directly induces the expression of cellulase and xylanase in *M. thermophila*. Ref. [127] has shown that the *HAC1* gene is required for the growth of *N. crassa* on cellulose.

Nevertheless, HacA and IreA can also suppress protein secretion, presumably to mitigate the protein load on the ER under ER stress conditions. Ref. [136] reported that ER stress induces transcriptional downregulation of the glucoamylase gene in *A. niger*. Consistently, HacA not only upregulates genes involved in the protein secretion machinery but also downregulates those encoding endogenous ER-client secretory enzymes in *A. niger* [133,137]. Likewise, HacA was reported to repress the expression of extracellular amylolytic enzymes in *A. oryzae* [124]. On the other hand, Ref. [14] proposed that mRNAs encoding ER client secretory proteins are digested by RIDD in *A. oryzae*.

The functions of HacA and IreA in filamentous fungi are also of significant clinical relevance. *Aspergillus fumigatus* is a major opportunistic pathogen causing invasive aspergillosis in immunosuppressed patients. Some publications have shown that deletion of the *hacA* gene impairs protein secretion, infectivity, and virulence in *A. fumigatus* [123,138,139]. However, according to Ref. [13], the *ireA* deletion mutant exhibited a more severe defect in virulence-related traits than the *hacA* deletion mutant in *A. fumigatus*, suggesting that IreA has a function other than *hacA* mRNA splicing. The Ire1 inhibitor 4μ8C can suppress UPR in *A. fumigatus* and may has potential as a therapeutic agent for aspergillosis [131,140]. Refs. [125,141] have shown that the *hacA* gene is required for morphogenesis, pathogenicity, and aflatoxin biosynthesis in *Aspergillus flavus*.

## 8. Conclusions and Future Perspective

Since *S. cerevisiae* is a widely used model organism for elucidating biological phenomena commonly observed in eukaryotes, the regulatory mechanism of UPR has been well elucidated in this yeast species. The activity and expression of two key factors, Ire1 and Hac1, are controlled in sophisticated manners to induce UPR with optimal strength and duration. Following studies in *S. cerevisiae*, the unconventional splicing of *HAC1* mRNA in response to ER stress has been demonstrated in a wide variety of ascomycete yeasts and filamentous fungi. Animal, plant, and basidiomycete species also carry mRNAs encoding bZIP-family transcription factors that are spliced by Ire1 [23,75,142]. However, discoveries made in *S. cerevisiae* cannot easily be generalized to other organisms. For instance, Ire1 is unlikely to splice a bZIP-family transcription factor mRNA in *N. glabratus* and *S. pombe*.

BiP cooperates with various co-factors, such as J-proteins, to maintain protein integrity in the ER. In *S. pombe*, Ire1 promotes RIDD, which specifically induces expression of BiP but not that of other proteins, including BiP co-factors [8]. On the other hand, Hac1 transcriptionally induces a number of genes, including those encoding BiP and its co-factors [46,47]. Therefore, upon ER stress, the complicated proteostasis machineries in which BiP plays key roles may be upregulated in *S. cerevisiae*, but not in *S. pombe*. In agreement with this, another ER-located molecular chaperone, calnexin, is likely to play more important roles in *S. pombe* than in *S. cerevisiae* [143]. These insights may explain why Hac1 is absent in *S. pombe*. Nevertheless, it is also possible that Hac1 expression in *S. cerevisiae* and RIDD in *S. pombe* lead to partially overlapping outcomes to alleviate protein load on the ER. Hac1 directly or indirectly downregulates the expression of some genes encoding ER client proteins transported to the cell surface in *S. cerevisiae* [47]. Similarly, many mRNAs encoding ER client proteins are degraded by RIDD in *S. pombe* [8].

Compared to *S. cerevisiae*, the intron excised by Ire1 is considerably shorter in a wide variety of organisms, including many ascomycetes. This suggests that the base-pairing between the 5′-UTR and intron shown in *S. cerevisiae HAC1* mRNA is not a widespread feature among species. Unspliced *HAC1* mRNA may be translated into a protein, whose function warrants further investigation, in certain ascomycete species other than *S. cerevisiae* [9,131]. In many *Saccharomycotina* and *Pezizomycotina* species, but not in *S. cerevisiae*, Ire1 likely performs RIDD in addition to *HAC1* mRNA splicing.

As a spherical unicellular organism that utilizes only low-molecular-weight nutrients, *S. cerevisiae* is unlikely to produce large amounts of proteins that traverse the secretory pathway, such as secretory digestive enzymes or cell surface proteins involved in hyphal formation. Therefore, the ER may be only minimally stressed under normal conditions in *S. cerevisiae*. We speculate that this is why *S. cerevisiae* has evolved multiple safeguard systems to suppress UPR under these conditions.

On the contrary, the ER in many filamentous fungal species is likely to be under heavy load, given that they abundantly secrete digestive enzymes and form hyphae. The same can be said for pathogenic yeast species, such as *C. albicans*. Therefore, Ire1 and *HAC1* in these species may have evolved to have more extensive roles than those in *S. cerevisiae*. For instance, given its ability to downregulate ER protein influx independently of gene transcription, RIDD appears to be an effective mechanism for coping with acute ER stress in highly protein-secreting cells. It should also be noted that Ire1 and *HAC1* have been reported to play multiple, as-yet-undisclosed roles in *K. phaffii*, which secretes proteins more abundantly than *S. cerevisiae* [9,87].

It also remains to be determined how Ire1 selectively executes *HAC1* mRNA splicing or RIDD in accordance with the nature of activation stimuli. In *S. cerevisiae*, while Ire1 is thought to exclusively catalyze *HAC1* mRNA splicing, the downstream gene induction profiles vary depending on the type of stress. The modulation of Hac1 function in selecting appropriate target genes is another intriguing research question.

UPR in ascomycetes holds great potential not only for basic biology but also for clinical and practical relevance. Given that Ire1 and Hac1 play crucial roles in the infectivity and virulence of pathogenic yeasts and fungi, the prominent Ire1 inhibitor 4μ8C has been proposed as a potential therapeutic agent for mycoses [110,131,140]. However, when applied to mammals for this purpose, pre-existing Ire1 inhibitors, including 4μ8C, can also inhibit the endogenous Ire1 ortholog. Moreover, off-target effects of 4μ8C have been reported [144]. We anticipate that new therapeutic targets for developing highly specific antimycotic drugs will be discovered by identifying yeast- and fungal-specific proteins or pathways that activate UPR. The UPR machinery has also been reported to increase the resistance of pathogenic yeasts and fungi against conventional antimycotic drugs [101,123,126]. Moreover, certain antimycotic drugs can induce ER stress in *C. albicans* [103,108,109]. Therefore, pathogenic yeasts or fungi may acquire drug resistance through genetic mutations that modulate UPR, which should be addressed in future studies.

UPR is also crucial for protein secretion in industrial yeasts and fungi, and Hac1 is frequently overexpressed to increase the secretory yields. To avoid the adverse effects of Hac1 expression, such as growth delay, its timing and expression levels should be carefully regulated. For this purpose, it may also be effective to control UPR levels not by directly regulating Hac1 expression levels, but by modifying the cellular machinery that modulates endogenous Hac1 levels, as done in Ref. [69]. In this context, the mechanisms controlling the activity and expression of Ire1 and Hac1 in industrial yeast and fungal species are important research topics. RIDD also warrants attention because Ire1 can degrade mRNAs encoding ER client proteins, including the target proteins to be produced. However, simply deleting the *IRE1* gene may not be an ideal strategy, as the loss of the UPR pathway compromises protein folding capacity in the ER. Therefore, overexpressing Hac1 in *IRE1*-knockout mutants would be an intriguing strategy for maximizing protein secretion.

Another important research topic for the future is the physiological outcomes of UPR and their significance. Although not discussed in detail in this article, transcriptomic analyses have revealed that Hac1 directly or indirectly controls a wide variety of genes, including those seemingly unrelated to the ER or secretory pathway, in many ascomycete species. This suggests that UPR not only enhances ER and secretory pathway functions but also remodels other physiological states, such as metabolic pathways. However, this point has not been fully elucidated, even in *S. cerevisiae*. Comprehensive omics studies, including metabolome, lipidome, and physiome analyses, would provide crucial insights into understanding the holistic consequences of UPR.

## Figures and Tables

**Table 1:** Functions of Ire1 in ascomycete species.

Species	*HAC1* mRNA Splicing	Other Roles
*Saccharomyces cerevisiae*	Yes	Not found
*Schizosaccharomyces pombe*	No *(HAC1* mRNA does not exist) [8]	RIDD (BiP mRNA is stabilized by RIDD) [8]
*Komagataella phaffii*	Yes	Maintenance of cytosolic protein integrity [9]
*Candida albicans*	Yes	Hyphal development and others; RIDD [10,11]
*Nakaseomyces glabratus*	No (*HAC1* mRNA exists but is not spliced) [12]	RIDD [12]
*Aspergillus* spp.	Yes	Virulence [13]; RIDD [14]

RIDD stands for regulated Ire1-dependent decay.

## Data Availability

No new data were created or analyzed in this study. Data sharing is not applicable to this article.

## Abbreviations

The following abbreviations are used in this manuscript:

DTT	Dithiothreitol
ER	Endoplasmic reticulum
ERAD	ER-associated protein degradation
HOG	High-osmolarity glycerol
LBS	Lipid bilayer stress
LUTI	Long undecoded transcript isoform
NGD	No-go decay
PDI	Protein disulfide isomerase
RIDD	Regulated IRE1-dependent decay
TSS	Transcription start site
uORF	Upstream open reading frame
UPR	Unfolded protein response
UTR	Untranslated region
